# Supplementary material for: ReVisE: Remote visualization environment for large numerical simulation datasets
Source: PLoS One. 2021 Jul 27;16(7):e0255030. doi: 10.1371/journal.pone.0255030 (PMC8315543; doi:10.1371/journal.pone.0255030)
Supplement: Attachment — Submitted filename: response_to_reviewers.pdf [file pone.0255030.s001.pdf]

1 To Editors

1.1

Q: Please ensure that your manuscript meets PLOS ONE's style requirements, including those for file naming. The PLOS ONE style templates can be found at...

A: Done

1.2

Q: We note that you have stated that you will provide repository information for your data at acceptance. Should your manuscript be accepted for publication, we will hold it until you provide the relevant accession numbers or DOIs necessary to access your data. If you wish to make changes to your Data Availability statement, please describe these changes in your cover letter and we will update your Data Availability statement to reflect the information you provide.

A: Yes, we have made all the data accessible. First of all, we have uploaded Revise's sources on Github: `https://github.com/deadmorous/revise`. It is distributed under AGPL-3.0 license. Also we have deposited all input data into protocols.io. Corresponding DOI are:

- Build ReVisE on Ubuntu:
  `http://dx.doi.org/10.17504/protocols.io.bruwm6xe`
- Prepare and run test on available dataset:
  `http://dx.doi.org/10.17504/protocols.io.bruzm6x6`

1.3

Q: We noted in your submission details that a portion of your manuscript may have been presented or published elsewhere: ...

Please clarify whether this publication was peer-reviewed and formally published.

If this work was previously peer-reviewed and published, in the cover letter please provide the reason that this work does not constitute dual publication and should be included in the current manuscript.

A: Yes, the article "Core Algorithms of Sparse 3D Mipmapping Visualization Technology" is peer reviewed and published.

This is the reference:

Orlov S., Kuzin A., Zhuravlev A. (2020) Core Algorithms of Sparse 3D Mipmapping Visualization Technology. In: Voevodin V., Sobolev S. (eds) Supercomputing. RuSCDays 2020. Communications in Computer and Information Science, vol 1331. Springer, Cham.
`https://doi.org/10.1007/978-3-030-64616-5_36`

The reasons requested are in the cover letter.

1.4

Q: Please amend the manuscript submission data (via Edit Submission) to include authors Alexey Kuzin, Alexey Zhuravlev, Vyacheslav Reshetnikov, Egor Usik, Vladislav Kiev and Andrey Pyatlin.

A: Done

1.5

Q: Please ensure that you refer to Figure 4 in your text as, if accepted, production will need this reference to link the reader to the figure.

A: The reference to Figure 4 inserted.

2 To Reviewer 1

2.1

Q: A major concern is the availability of the software. It is stated that the source code will be made available at GitHub as soon as the paper is published. However, as it is increasingly acknowledged by the scientific community, the source code of any scientific software funded by public institutions and aimed at open source should be available for review. Besides, having an instance with a simple visualization example in a server as demo with a link provided to the reviewers (and later for potential users would also be beneficial) will make a stronger publication.

A: The source codes of Revise are now available at GitHub under AGPL-3.0 license: `https://github.com/deadmorous/revise`. Also all input data is deposited into protocols.io. Corresponding DOI are:

- Build ReVisE on Ubuntu:
  `http://dx.doi.org/10.17504/protocols.io.bruwm6xe`
- Prepare and run test on available dataset:
  `http://dx.doi.org/10.17504/protocols.io.bruzm6x6`

2.2

Q: Regarding technical details of implementation and deployment, it would be useful to add in Fig. 8 the programming languages of the main software components. Other helpful additions could be mentioning what are the dependencies if there are any and an estimate of the percentage of new code that was implemented or the one re-used (the authors mentioned that it is based on source code of OpenGL fragment shaders found in the Visualization Library).

A: We fixed Fig. 8.

Presently Revise does not depend on any external modules, besides trivial ones (such as Qt, googletest). It also depends on VisualizationLibrary, which is used as external library and is loaded as Git submodule. Presently Revise is

intended to support two implementations of rendering: with CUDA and with OpenGL. The article does not span the second one because it is not developed well now and, probably, will not be developed in the future due to some difficulties when multiple GPUs used and due to absence of advantages over CUDA implementation. Revise depends on VisualizationLibrary when it is built in OpenGL mode because the rendering works via VL API. The dependency on VL in CUDA mode is rudimentary.

When we write that our implementation of rendering is based on source code of shaders of VL we mean that we refer to algorithms used but the code is almost fully re-written. VL rendering is OpenGL-based and written in GLSL, when our rendering is CUDA-based so it has to be written from scratch as CUDA kernels. The VL implementation generally contains classical algorithms, such as volume raycasting and we used this implementation as reference. The only thing that can be treated as some sort of copying from VL is Blinn lighting implementation. Here is the comparison of these both sources:

Revise:

`https://github.com/deadmorous/revise/blob/master/src/s3dmm_cuda/cuda_lighting.hpp#L27`

`https://github.com/deadmorous/revise/blob/master/src/s3dmm_cuda/cuda_lighting.hpp#L54`

VL:

`https://github.com/MicBosi/VisualizationLibrary/blob/master/data/glsl/volume_raycast_isosurface_transp.fs#L44`

`https://github.com/MicBosi/VisualizationLibrary/blob/master/data/glsl/volume_raycast_isosurface_transp.fs#L66`

But it is worth noticing that Blinn lighting is well-known classical algorithm with well-known implementation.

2.3

Q: The authors claim that tests show that ReVisE outperforms other systems in some cases, especially for large time-dependent datasets. However, the comparison with other systems does not seem quantitative. Even if numbers are from the respective publications of the other tools, a comparison table indicating dataset, hardware, execution time and some comments specific to each system would be preferred.

A: A comparison of volume rendering of ParaView and ReVisE added.

2.4

Q: Minor suggestions are made to improve the manuscript:

- Results: please add details of the deployment used for the tests. Where all servers in the same computer? **A:** ReVisE can be deployed on a Linux system by building it from the source code, or by using a docker image. See `https://github.com/deadmorous/revise#installing-revise` and

`https://github.com/deadmorous/revise#docker`. In addition, an installation procedure is described in this protocol: `http://dx.doi.org/10.17504/protocols.io.bruwm6xe`

- Line 534: GPU memoy → GPU memory **A:** Done
- Line 550: didn't → did not (too informal otherwise) **A:** Done

3 To Reviewer 2

3.1

Q: The paper introduces a new visualization system, called ReVisE, that aims at remotely visualizing large—not of the reviewer numerical simulation—datasets. I have appreciated to review this article, that has an overall interesting content, and that takes the pre-processing approach in order to solve a task that is otherwise not reachable at the scale of nodes the author intend to work with in real time. We have nonetheless to wait line 8 to understand the kind of data that are manipulated, also something like "for large numerical simulation datasets" in the title and abstract would immediately reduce the scope of the article, as the purpose is not to visualize other kind of large datasets such as corpus of texts.

A: Done

3.2

Q: In the introduction, the lack of available visualization software that scales up is emphasized (l.13) to motivate the introduction of their system or with performance up to 10^6 nodes. If the scaling issue is tackled clearly in the rest of the article, does the proposed platform intended to be available Open Source, is there a Demo somewhere or at least a video of the platform? The related work relies more on other softwares / products, than on what are the existing technologies, what is the state of the art this Research article is linked to.

In order to help relates this article to existing work, references such as BEYER 2014 State of the Art in GPU Based Large Scale Volume Visualization or Wang 2020 Portable interactive visualization of large-scale simulations in geotechnical engineering using Unity3D, which introduces not only the different softwares but also the different technologies to motivate deeply their approach, could be used. A beginning of argumentation and a clear introduction of the plan should be made at the end of the introduction.

A: Added reference to BEYER 2014; added three paragraphs at the end of the introduction that

- introduce main ideas from BEYER 2014;
- classify ReVisE in terms of categories defined in BEYER 2014;

- outline further sections of the paper.

3.3

Q: Sections, paragraphs and subparagraphs at least should be numbered, as it would really simplify the reading, especially when forward references are made as the size of the police is not sufficient to make a clear distinction between sub-paragraphs and sub-sub-paragraphs. When pointing to Forward or Backward reference in your article, you should refer precisely.

A: We make the manuscript according to the template of the journal and, unfortunately, it does not have sections numbering. We added referencies to the sections, where needed.

3.4

Q: The task to be solved should be clearly stated as such in the introduction and related to the research question the author want to solve; formulating it properly, would induce the steps to achieve to properly evaluate the proposed platform, in comparison with other existing platforms.

A: In order to address and resolve those issues, we have tried to improve problem formulation in the "Introduction" section. We also added more comparison results in the "Results and Evaluation" section.

3.5

Q: The article appears to be an extension of the already published article [12] (from line 66 to line 315). It should be stated as such both in the abstract and in the introduction. The new contributions of this article have to be clearly stated from the introduction. To help clarify this point, the paragraph Sparse 3D mipmapping, should not only said that the sparse mipmapping has been proposed in [12] but also mention that the whole paragraph reintroduce the concepts for the sake of clarity. Having an Arxiv version of [12] would definitely help the interested reader. When full paragraphs (l.224 to 248) are taken from [12] they should be quoted and referred as such. Figures from [12] (Fig.3 that corresponds to Fig. 2 and 5 of [12], Fig 4 that is Fig 1 of [12], Fig 5 that is Fig.7 of [12]) cannot be reproduced as such for evident copyright reasons: they should either differ or be cited from [12]: in this case, authors should have the consent of Springer for reusing them in another article.

A: The section Sparse 3D mipmapping has been refactored and made shorter with more tight references to [12]. It is stated that it retells article [12], but does not replace, so the reader has to refer to [12] for more details. According to this logic Figures 3, 4 and 5 are removed in order to not duplicate corresponding figures from [12].

3.6

Q: L.242 to 270: How to compare two things that will not be comparable, with a part of pre-processing with the ReVisE and other system that are synchronous? The only way is to compare at least the full process and show what are the gains / costs of making it asynchronous. The other point, is that the scalar field has to change over time, also how are you going to take that into account if it is in the part of the pre-processing? This paragraph lets understand there is still work to do on that (l.269-270), which would explain that the pre-processing is not taken into account (l.244-245); and in this case how do you evaluate a reasonable duration (l.245)? How do you compare it to existing frameworks? l. 266 to 268: are there corroborating results to assert the advantage of the proposed algorithm as [12] does not show any baseline for comparing? Moreover the algorithm for stage (5) should be given.

A: We think, that on comparison of the speed of interactive rendering between ReVisE and other systems, we do not need to take into account preprocessing time in ReVisE. One should compare the time of the rendering of the scene: this time is important for the user because it determines the level of convenience of usage of the system for interactive visualization. Thus, this time may serve as quantitative characteristic of convenience of use of the system. Reduction of this time can be achieved, on the one hand, by speeding up all stages, and also, by transferring some operations to offline preprocessing. In ReVisE we use, first of all, the second approach.

Changing of the scalar field over the time is also allowed. Visualization dataset consists of metadata that is common for all time frames and arrays of sparse fields, calculated for each time frame. An evaluation of visualization dataset is performed at the preprocessing stage.

ReVisE preprocessing times have been included into the new version of the paper. Also, it turns out that to gain best performance with Paraview, the original dataset has to be preprocessed as well (otherwise, the animation performance is completely unacceptable). We have compared preprocessing times and visualization dataset sizes for ReVisE and Paraview.

We decided not to present the algorithm for stage (5) because it has already been given in our published paper [15]. Presenting the algorithm in this paper would increase the intersection of paper content with content published elsewhere, which is not desirable. As follows from the algorithm, it is a single-pass one, and should scale good enough as dataset size grows; of course, the comparison with other algorithms needs to be done and will be done as soon as we implement our algorithm and present it in a future paper.

3.7

Q: The new contributions of the article comes from line 316 and forward, include the introduction of the general architecture of the visualization system, moving to the engineering part of the project, mostly descriptive of the implementation; the level of details is largely sufficient to follow.

A: Does not require answer

3.8

Q: Results part (and I would suggest to rename this part Results and Evaluation):

To evaluate the performances of the proposed system, the proposed baseline in Comparison with other systems relies mainly on open systems, and particularly Paraview. As it is mentioned Paraview does the pre-processing on the fly, so it is going to be hard to compare two processes that do not rely on the same basis. In order to keep this baseline, results on preprocessing time and visualization processing time should be gathered in one table for both systems. If they are equivalent for the different datasets, then we can conclude that the one that has the pre-processing is going to outperform the one that has to recompute each time. A Table with figures for each stage is expected at that level for at least ReVisE and the baseline, for each proposed dataset and for the same level. Additionaly, l. 403 (preprocessing times have also been measured) contradicts l. 245, and no figures are given.

A: We have renamed Results to Results and Evaluation.

We restyled section Discussion (subsection Comparison with other system), therefore there is comparison of animation frame rate for ParaView and Revise. We have added preprocessing time of Revise into the table.

First of all the time of animation is compared. As one can see an animation in Revise has higher rate than in PraView's one. Of course, one can add preprocessing time to Revise's animation, but as one can see in this case it would be a little bit faster.

Also it should be noticed that ParaView also requires preprocessing. We have to transform original dataset into pvti format in order to provide higher rate in ParaView. As it has mentioned in the article, it requires about 4 hours, which is a real preprocessing time in ParaView.

It should be noticed that this comparison relates to the playing of time history. If one does pan/zoom/rotate in ParaView without changing time frame the volume rendering is performed very fast.

3.9

Q: In the Visualization performance (first occurrence l.428, as a second paragraph l.557 is named the same) the results are centered on the presented system; some tables presenting the datasets with the number of nodes at each level would be an asset (l.435-440). The approach is interesting.

A: The table with visualization datasets parameters added. Also the second subsection with name Visualization performance is united with Future work.

Q: For helping the reading l.465 to 475 should be put before l.457 to 464 in order to avoid a forward reference on l.456.

A: Corresponding paragraphs are swapped.

Q: In the Visualisation quality, there is the need to evaluate it properly: which task has to be solved? which metric is used to show the improvement on the quality and how do we compare this quality to a baseline (what would have been generated by Paraview)? Only experts of the field could answer about the quality of such visualization and respond to what is asserted in lines 487 to 490, as in fact to restrain to level 3 might not be as assumed in the introduction the level of quality the experts would need to have. This evaluation should be done by mixing those visualization with visualization generated with Paraview.

A: As far as of the quality of volume rendering, let us notice first that the best possible level of detail is achieved as soon as the texel size of the 3D texture does not exceed the size of a pixel on the screen. Because of this, the effective texture resulution of 2048 that we have in the cascade dataset ensures the best possible level of detail on a typical FullHD dispay (1920 x 1080 pixels), unless the view is zoomed. Further, it might turn to be good enough to have texel size larger than the pixel size, if the gradient of the field being visualized is not too high. In general, the desired level of detail in the 3D texture depends on the problem at hand and, in particular, on mesh element size (which is respected by the ReVisE preprocessor). We would like to emphasize that in ReVisE user has full control on the level of detail in the visualization dataset, because the maximal number of block level is explicitly set at the preprocessing stage, which together with block depth determines the effective resolution of the 3D texture.

Also there is the following observation about datasets from the CFD area: those datasets are often large due to mesh refinement at the boundary, which is necessary for the computation; however, it is typically normal to ignore those boundary layers during the visualization. Anyways, it is up to the user how to choose the desired level of detail by setting the mesh refinement parameter α, the boundary refinement parameter, and the limitation on the number of levels.

3.10

Q: In the paragraph Comparison with other systems, you mention l.504 "According to our experience" for the better interactivity: this is very subjective and need to be quantified. Particularly, I would have expected to see results on the number of fps rendered with Revise compared to other systems. Once again a well designed Table with different criteria for comparing the systems would be welcome.

A: A comparison of frame rates between ParaView and ReVisE are added for hump model.

3.11

Q: I would put the second paragraph Visualization performance in Future work (more than Further work) as from line 564 it concerns only Future work.

A: Visualization performance subsection is moved to Future work.

3.12

Q: A last question has to be raised with the final sentence: why would not have started by integrating the ReVisE in the Paraview? I think a different formulation can be found for this point.

A: In the beginning of our work on the project, we tried a different approach to the visualization. One of our students was developing a plugin for Paraview. Lessons learned were that (a) it is quite difficult to follow all the rules of Paraview plugin development, because of many things to be taken into account; (b) Paraview is a large system, and in order to extend it properly with plugins, one needs to learn Paraview source code; (c) Paraview visualization pipeline might have performance bottlenecks that are not known in advance, and struggling against those bottlenecks would take up considerable time. But what we really wanted was to concentrate on the proof-of-concept implementation of our approach, rather than to learn the details of Paraview implementation. To achieve that goal, it was significantly simpler to start from scratch and have full control over literally any aspect of the system, than to try extending Paraview and overcome unexpected problems we are faced to. And only after our approach proves feasibility, it worth trying to build it into Paraview; in this case, the existing ReVisE implementation serves as a reference for performance estimation and helps to control that the Paraview implementation is being done properly.

3.13

Q: Additional remarks: (a lot)

Direct style (for instance l.60-61), imperative forms (l.110, l.281 for instance) should be avoided in a scientific article.

A: Fixed

Statements should be accompanied by proovable arguments; particularly when coming to evaluation this is definitely needed. I would have expected the visualization performance to be compared to existing softwares, and particularly to Paraview as far as the number of nodes are comparable (maybe for instance with the hump dataset). It would have included in this case the preprocessing stage and the visualization stage, as it has been partially done in [12], showing the gain that we can expect from the new platform. Coming back to the assertion made on line 60-61 there is no proof (i.e. no comparison on an A/B testing for instance) of the visualization made. A baseline would be again to simplify the problem in a first instance to make two comparable visualization one obtained by Paraview for instance and one obtained by your method, and then scaling, and showing what happens. A video comparing the two could also be an asset.

A: We added comparison of animation rate in ParaView and ReVisE. The preprocessing time is also taken into account. See Comparison with other systems subsection.

Q: A particular attention to articles (a and the) should be given, particularly when they are missing (for instance l.339: it uploads the image to the shared

memory; l.343: the video streaming service checks the shared memory cotents).

A: We tried to take it into account.

Q: Bullets should be avoided in an article (it is not a presentation); a table is often a good replacement (l 405 to 437 and l.420 to 427)

A: Fixed

3.14

Some remarks on Figures:

Q: All the figures come at the end of the article and are not included in proper way in the article, which makes things difficult to follow.

A: This is requirement of the journal to place all figures separately from the main text.

Q:Figure 1: I would let the mesh behind in B (as it is done in C). Moreover, the frame used for the quadtree should be marked as such in A, just to be sure that all Figures from A to F are at the same dimension of representation (which does not seem to be the case).

A: In all subfigures the scale is the same. The mesh behind the quadtree has been added. Notice that the bounding box of the quadtree is greater than that of the original mesh by some "padding" and is obtained by including points $\mathbf{r} + \mathbf{n}s$, where $\mathbf{r}$ is the position of a node of refined boundary mesh, $\mathbf{n}$ is the unit vector of outer normal vector to the boundary face, and s is the size of the refined boundary face. Since in subfigure E we use 10x boundary refinement, and in subfigure B we use no (1x) boundary refinement, the quadtree in B has 10x larger padding than in E. It is correct and I am not sure that we need to clarify it in the text. The need for the padding yields from the need to have a field whose zero-valued isosurface approximates domain boundary.

Q: Figure 2: I think the mesh chosen for illustrating a bit hard to follow and would let it by transparency under the different quadtrees generated.

A: Done

Q: Figure 3: I would add colors to help following the octree, or / and draw the graph tree corresponding.

A: The Figure was removed due to restyling of Sparse 3D mipmapping section.

Q: Figure 4: having the mesh by transparency under the quadtrees generated would help.

A: The Figure was removed due to restyling of Sparse 3D mipmapping section.

Q: Figure 5: It differs only from one node from Springer. A legend indicating nodes signification directly on the Figure it self would help (and not in the Legend below the Figure). The Legend below figure 5: the second B should be C.

A: The Figure was removed due to restyling of Sparse 3D mipmapping section.

Q: Figure 8: two inversions: Numerical solution (oi inverted) and Web server: visualization controller (rt inverted)

A: Fixed

Q: Figure 9: ReVisE web interface and not ReVesE in the legend

A: Fixed

Q: Suggestions of figures: A figure similar to Figure 3 of [12] would clarify lines 126-127.

A: Now we reduced the section Sparse 3d mipmapping and it is more dependent on content of [12]. Therefore in order to not overload the article's content it is intended that the reader will refer to [12] for more information.

Q: A figure might help to clarify l.154 - 162.

A: Added Fig 3. An example of quadtree and corresponding metadata.

3.15

Mispelling / other remarks:

Q:l.403: preformance instead of performance

A: Fixed.

Q: l.345: what information does the sentence bring?

A: Removed.
