# Supplementary material for: ReVisE: Remote visualization environment for large numerical simulation datasets
Source: PLoS One. 2021 Jul 27;16(7):e0255030. doi: 10.1371/journal.pone.0255030 (PMC8315543; doi:10.1371/journal.pone.0255030)
Supplement: Attachment — Submitted filename: response_to_reviewers_2.pdf [file pone.0255030.s002.pdf]

1 To Reviewer 2

1.1

Q: Introducing the baseline comparison suggested during the first review improves the Evaluation part. Nonetheless, from your comments: "It should be noticed that this comparison relates to the playing of time history. If one does pan/zoom/rotate in ParaView without changing time frame the volume rendering is performed very fast.", I have a question: What does happen with Revise in the pan/zoom/rotate case? Do you have additional evaluation of this point compared to paraview, such that the reader can understand all the advantages and potential drawbacks, are generally there is not only one side of the coin.

A: A paragraph has been added to sec. 4.2 "Comparison with other system", started with words "As a preliminary test, interactive pan/zoom/rotate operations have been tested in ParaView and ReVisE ...". The paragraph gives some basic information about the performance of pan/zoom/rotate operations without animation, sufficient to make some conclusions, but without thorough measurements, which is problematic due to a number of reasons (wide range of reported fps values, the dependency of fps on the quality of network connection, on opacity distribution of color transfer function, on visualization settings, on hardware configuration, etc.)

1.2

Q: More anecdotically, preparing the dataset to be in an acceptable format for the system it is going to be read is required but I would not labelled it as a pre-processing stage as you need to do for ReVise.

A: Well, we could drop any mentioning of the fact that data needs to be preprocessed for ReVisE, and it consumes some computational resources, simply specifying that the data has to be in our octree-based format. But we think it is better to leave the things as they are, due to the following reasons.

- ReVisE can only visualize data in one specific format; that format is not a natural choice for the output of numerical solution, at least for problems we have dealt with.
- ParaView can accept a variety of data formats, some being a natural choice for the output of numerical solvers (e.g., CGNS).
- For some techniques of visualization, such as volume ray casting, the performance of ParaView is very sensitive to input data format. For example, the "image" format is good, whereas a format based on structured/unstructured grid data is not. In fact, ParaView is doing preprocessing "on the fly" for that use case, due to which the performance drops

critically. We think that the illusion that one can supply data for visualization in any format is quite misleading, because the preprocessing stage is hidden "under the hood". The presence of explicit grid-to-image data conversion in ParaView pipeline is also not very good, because users do not really avoid spending time waiting while that conversion is done during their interactive session with the visualization system. That is the primary point why we want to do a clear separation between preprocessing (before user even starts spending data on the interactive session) and visualization.

1.3

Q: Additionally, I would have expected a figure similar to Figure 11 to compare the two renderings (the one achieved with paraview and the one achieved with Revise for the hump model.

A: Done, but for the `cascade` model. We wrote a tool, named `to_cgns` and made part of the repository, that converts any format readable by ReVisE preprocessor to the CGNS format (though, the current version only takes the first time step). Readers can now compare images generated by ReVisE against similar images generated with ParaView (new Figure 12 has been added). Besides, ReVisE isosurface shown in Figure 12 at the highest level of detail is also shown in Figure 11 at different levels of detail, so readers can see how close the images at different levels of detail are to the "ground truth" ParaView image of isosurface computed from mesh data. That's why `cascade`, not `hump`. We had to replace Figure 11, mostly because with the old one it was difficult to have the same camera transformations in ParaView and ReVisE. And thank you for requesting the figure with the comparison: during the work on it, we have revealed and fixed a bug in the ReVisE preprocessor!

1.4

Q: Additionally, I would put this evaluation with the corresponding dataset on a downlable virtual machine for both Paraview and Revise system in order they can be run out of the box for reviewers and readers.

A: We have prepared a downloadable Docker image that contains a ReVisE installation. The image requires CUDA 11.1 or higher to be installed on the host machine. We did not create a VM, for example, VirtualBox, because of the essential dependency on CUDA: the machine, anyway, would depend on the host OS.

Our new protocol `http://dx.doi.org/10.17504/protocols.io.bv5hn836` describes how to install and use the Docker image. The protocol also refers to our previous protocol `http://dx.doi.org/10.17504/protocols.io.bruzm6x6` describing the preparation and running test datasets.

The docker image does not contain ParaView, but ParaView can be installed on the host machine. In order to display our datasets in ParaView, one should convert the dataset to the CGNS format. To do so, one should use the `to_cgns` program, which is included in the Docker image. Please see comments to protocols mentioned above for information on how to call `to_cgns` and run `pvserver` when the Docker is running.

1.5

Q: l. 15: you start by speaking of NVIDIA Index and Sight. NVIDIA Index is then presented in details in paragraph in l. 28 ... but no more mention of Sight.

A: Presentation of Sight added.

1.6

Q: l.59-61: I would reformulate the answer to the question in a less assertive mode, because nothing is proven at this stage, by putting: In this paper, we are going to show that it is in fact not needed, otherwise the answer asks for explanation, which is mainly what you are going to present in the article.

A: Fixed

1.7

Q: I know you have mentioned that it is an editor constrain but Numbered sections are definitely needed to refer to Sections and Subsections (the Editor mentioned I should put it in my review comments). Additionally, to refer to a Section or Subsection, the rules is similar than the one applied for figures, i.e. the section name should be prefixed by Section / Subsection.

A: Numbering added

1.8

Q: Avoid of ... of... in a sentence: it is often avoidable by reverting (3 in one sentence line 95 - 96 for instance, and also l.401, but certainly on other places)

A: Done (rephrased in many places).

1.9

Q: Presenting an additional idea goes with three em-dashes: `https://www.uhv.edu/university-college/student-success-center/resources/a-d/dashes-use/`

#:~:text=There%20are%20actually%20three%20different,writing%20to%20an%20old%20friend. This would be appropriate in l.111-112, l.116-117, l.312, l.418-419, l.497 for instance.

A: Sorry, we failed to understand what you meant. Could you please give an example of original and corrected text? Also, does "three em-dashes" in your remark mean the same as "3-em dash" in the web-page you referred? In that page, "3-em dash" is a kind of placeholder, not even a real dash used in typesetting...

Q: Websites reference should never been put in a bibliography section but as bottom page notes, unless it is a requirement of the Editor (ref 30, 31, 1, 5, 6) as they are not proper bibliography references.

A: These references moved to footnotes

Q: Some bullet (or telegraphic) style paragraphs remain: it is never a good idea to put more than one semi-column in a sentence. Additionally, each sub-sentence should have a subject and verb in this -case. (l. 17-20, l.86-91, l.73-77, l.296-299)

A: l.17-20: extended and split into three logically related parts. l.86-91: Described the structure of next section with separate sentences and in more detail, referring to the sections by their numbers. l.73-77: replaced semicolons with commas. l.296-299: no semicolons were there, no changes made. In addition, semicolons are replaced with commas in Figure 7 caption.

1.10

Q: l. 158: roams instead of walks is maybe more appropriate

A: We are not sure that "roams" is more appropriate than "walks". At least authors have happened to find usages of the verb "walk" as "iterate through all elements in a collection"; though, the collection was more often a tree than a list. For example, googling "walk octree" (with quotes) gives 10 results, and googling "roam octree" gives 4 results, all not relevant because ROAM is an abbreviation. To make the text more clear, we replaced "walks" with "iterates".

1.11

Q: l. 162 - l.164: on the one hand is needed in place of importantly. End of line 161: I would add: The element size s is bounded between two values s_min and s_max. On the one hand, the smallest value s_min ...

A: Done.

1.12

Q: l. 177: what is a ”typical” visualization? As typical, depends mostly on the level of details you want to achieve.

A: Replaced “for typical visualization” with “in many practical cases”. These restrictions are not hard-coded in ReVisE; the user decides which values to choose. But we mentioned the limitation of the total octree depth as something reasonable, because in our early experiments without any limitation and with $\alpha = 0$ we sometimes obtained huge octrees of unpredictable sizes. With total depth limitation, one can be sure that the dataset size is below certain value, even with $\alpha = 0$.

1.13

Q: l. 233 ... maybe: The interested reader can refer to [] on the way of addressing such elements and for further details. [13] should be accessible on Arxiv, as it is intensively refered.

A: Line number seems to be incorrect. The closest reference to [13] in line 246; there are also many other places referring [13]. We agree that the paper is intensively referred. However, putting it in its final form to Arxiv is explicitly disallowed by Springer’s consent to publish, signed by authors. The same document allows preprints to remain on Arxiv; but is adding a preprint to Arxiv after publication in Springer a common practice? Maybe we should order retrospective open access instead? Can you give any advise?

Q: l. 685: to distribute

A: Last numbered line, before References, has number 637. If the numbering is extended to the list of references, line 687 contains reference [19]: “Lefebvre S, Hoppe H. Perfect Spatial Hashing. ACM Trans Graph.”. What did you mean? Most likely, line number is incorrect.

Q: Articles should be checked, particularly when nouns are singular (here an article is often needed) as their non usage—named Zero-marking—is mostly dialectal english or reserved to particular cases.

A: Hopefully fixed this issue, at least partly.

2 To Reviewer 3

2.1

Q: Where the paper is weaker is in the comparison section. The authors correctly identify the performance bottleneck in ParaView as the IO time. They should not have stopped there and should have explained more thoroughly how ReVisE overcomes this issue.

Furthermore, it would have been good to better compare interaction performance rather than mainly focus on animation. Similarly for IndeX and OSPRay comparisons. Although this is not necessary to accept this paper IMO.

A: A paragraph has been added to sec. 4.2 "Comparison with other system", started with words "As a preliminary test, interactive pan/zoom/rotate operations have been tested in ParaView and ReVisE …". The paragraph gives some basic information about the performance of pan/zoom/rotate operations without animation, sufficient to make some conclusions, but without thorough measurements, which is problematic due to a number of reasons (wide range of reported fps values, the dependency of fps on the quality of network connection, on opacity distribution of color transfer function, on visualization settings, on hardware configuration, etc.) IndeX and OSPRay have not been investigated, mostly due to the lack of human, time, and hardware resources (the equipment of our laboratory had to be moved to a different location right after the second paper review and we have managed to set up and power-on again the "Tesla" only a few days ago).

Also, added new Figure 12 that compares rendered images in ParaView and ReVisE.

As of a more thorough explanation how ReVisE overcomes the IO bottleneck, we can say that ReVisE has been specifically designed to be IO efficient from day 0, while in the case of ParaView, in our opinion, the main idea was the flexibility and potentially a wide range of general functionality. ReVisE dataset storage format ensures the ability to read data contiguously, without having to perform many seek operations. The amount of data to be read varies, depending on the desired detail level, which allows to generate preliminary low-resolution image very fast. A few words about that are at the end of subsection 2.1.3. Also, we have some experience with reading data in some formats supported by ParaView using the VTK library. Although we did not measure the performance of read operations, there was a feeling that the data could have been read much faster than it really was with VTK.

We added a paragraph containing the text close to the above (lines 606–617).
