# Supplementary material for: ReVisE: Remote visualization environment for large numerical simulation datasets
Source: PLoS One. 2021 Jul 27;16(7):e0255030. doi: 10.1371/journal.pone.0255030 (PMC8315543; doi:10.1371/journal.pone.0255030)
Supplement: Attachment — Submitted filename: response_to_reviewers_3.pdf [file pone.0255030.s003.pdf]

1 To Reviewer 2

1.1

Q: Presenting an additional idea goes with three em-dashes: `https://www.uhv.edu/university-college/student-success-center/resources/a-d/dashes-use/#:~:text=There%20are%20actually%20three%20different,writing%20to%20an%20old%20friend`. This would be appropriate in l.111-112, l.116-117, l.312, l.418-419, l.497 for instance.

A: Sorry, we failed to understand what you meant. Could you please give an example of original and corrected text? Also, does "three em-dashes" in your remark mean the same as "3-em dash" in the web-page you referred? In that page, "3-em dash" is a kind of placeholder, not even a real dash used in typesetting. . .

Reviewer: The reference to "three em-dashes" applies to a Latex option. The reviewer is suggesting that each time you have side comments in brackets as for instance in this sentence (line 123-124 revised version):

"Suppose there is an original mesh (unstructured or structured, consisting of tetrahedra or hexahedra — does not matter), and a scalar field specified at mesh nodes."

you should use the "three em-dashes" option of Latex (`\textemdash`) and write:

"Suppose there is an original mesh—unstructured or structured, consisting of tetrahedra or hexahedra, it does not matter—, and a scalar field specified at mesh nodes."

It is ultimately your choice. This is only a suggestion.

A: We have applied this rule in a number of places: l.111–112, as above but without comma after em-dash, l.309–310, l.412–413, Fig 9 caption, l.517 (line numbers correspond to the version before second review).

Also, spaces around the em-dash have been removed everywhere in the text, similar to the suggested example (in Russian, which is the authors' native language, spaces around em-dash are mandatory; for English, that can vary as we have found in `https://www.thepunctuationguide.com/em-dash.html`).

1.2

Q: l. 233 ... maybe: The interested reader can refer to [] on the way of addressing such elements and for further details. [13] should be accessible on Arxiv, as it is intensively refered.

A: Line number seems to be incorrect. The closest reference to [13] in line 246; there are also many other places referring [13]. We agree that the paper is intensively referred. However, putting it in its final form to Arxiv is

explicitly disallowed by Springer's consent to publish, signed by authors. The same document allows preprints to remain on Arxiv; but is adding a preprint to Arxiv after publication in Springer a common practice? Maybe we should order retrospective open access instead? Can you give any advise?

Reviewer: Regarding your questions about publishing rights regarding your Arxiv reference. The answer of the reviewer is as follows:

"You should check the date of the end of the embargo period (generally 12 months), as you could have it on an institutional website; anyway normally you are authorized on a personal website (it should be on the agreement). You can refer to: `https://www.springer.com/gp/open-access/publication-policies/self-archiving-policy`".

A: We have read about Springer's Self-archiving policy and agree to publish the Author's Accepted Manuscript (AAM) on ArXiv, as soon as 12 month period after first publication ends (i.e., in the very end of year 2021). At the moment, Springer's Consent to publish is not totally clear for us, therefore we plan to ask them about details of archiving of AAM to ArXiv. Also we would prefer to upload the AAM on ResearchGate, but it looks like Springer's policy does not allow to make it public.
